# Identifying genes associated with obstructive congenital heart defects using a family-based genetic random field method

**DOI:** 10.1016/j.xhgg.2026.100648

**Published:** 2026-07-06

**Authors:** Manyan Huang, Nianjun Liu, Stephanie M. Ware, John S. Witte, Bruce D. Gelb, Wendy N. Nembhard, Martha Werler, Meredith M. Howley, Lorenzo Botto, A.J. Agopian, Omobola O. Oluwafemi, Mary M. Jenkins, Lynn M. Almli, Andy Olshan, Paul A. Romitti, Gary Shaw, Ming Li, the National Birth Defects Prevention Study

**Affiliations:** 1 Department of Epidemiology and Biostatistics, Indiana University Bloomington, Bloomington, IN 47405, USA; 2 Department of Medical and Molecular Genetics, Indiana University School of Medicine, Indianapolis, IN 46202, USA; 3 Department of Epidemiology and Population Health, Stanford University, Stanford, CA 94305, USA; 4 Department of Biomedical Data Sciences, Stanford University, Stanford, CA 94305, USA; 5 Mindich Child Health and Development Institute and Department of Pediatrics, Icahn School of Medicine at Mount Sinai, New York, NY 10029, USA; 6 Department of Epidemiology, University of Arkansas for Medical Sciences, Little Rock, AR 72202, USA; 7 Department of Epidemiology, School of Public Health, Boston University, Boston, MA 02118, USA; 8 Birth Defects Registry, Bureau of Environmental and Occupational Epidemiology, New York State Department of Health, Albany, NY 02237, USA; 9 Department of Epidemiology and Biostatistics, College of Integrated Health Sciences, University at Albany, Rensselaer, NY 12222, USA; 10 Division of Medical Genetics, Department of Pediatrics, University of Utah School of Medicine, Salt Lake City, UT 84132, USA; 11 Department of Epidemiology, School of Public Health, University of Texas Health Science Center at Houston, Houston, TX 77030, USA; 12 Division of Birth Defects and Developmental Disabilities, Centers for Disease Control and Prevention, Atlanta, GA 30333, USA; 13 Department of Epidemiology, University of North Carolina, Chapel Hill, NC 27599, USA; 14 Department of Epidemiology, College of Public Health, University of Iowa, Iowa City, IA 52242, USA; 15 Division of Neonatal and Developmental Medicine, Department of Pediatrics, Stanford University, Stanford, CA 94305, USA

**Keywords:** congenital heart defects, family-based association study, rare variants, genetic heterogeneity

## Abstract

Congenital heart defects (CHDs) are the most common major congenital anomalies and the leading cause of infant mortality attributable to birth defects. Although substantial progress has been made in the identification of single-nucleotide variants associated with CHDs, the genetic etiology of CHDs remains largely unknown. While many studies have focused on common variants, efforts are underway to identify rare variants with heterogeneous effects on CHD development. We applied a family-based genetic random field (FGRF) method to buccal cell DNA specimens collected for the National Birth Defects Prevention Study, including 1,123 case families and 1,481 control families. We and others previously showed that the FGRF method was able to identify rare genetic variants associated with disease outcomes in the presence of disease heterogeneity. In this study, we conducted gene-based association tests for 24,270 genes and identified that *SLC44A2* was significantly associated with obstructive CHD risk after Bonferroni adjustment for multiple testing, representing the most robust signal observed in our analyses, while other phase-specific findings require further validation. Our subsequent gene set enrichment analyses supported the likely role of this gene in the biological pathways relevant to CHD development. Our study is complementary to existing genome-wide association studies on CHD risk, providing additional insights into the genetic etiology of these birth defects.

## Teaser

A genetic association study among participants of the National Birth Defects Prevention Study identifies genes associated with obstructive congenital heart defects by utilizing a biostatistical method accounting for genetic heterogeneity and cumulative effects of rare and common variants.

## Introduction

Congenital heart defects (CHDs) are the most common major congenital anomalies and the leading cause of infant mortality within the first year of life.^1^ In the United States, CHDs affect about 1% of live births and account for about 30% of deaths during infancy.^2^^,^^3^^,^^4^^,^^5^ Advances in diagnosis and clinical care have substantially improved survival, and many individuals with CHDs now live into adulthood. However, some may experience long-term health impacts and are affected by different kinds of co-occurring conditions such as extracardiac birth defects, which affect approximately 13% of individuals with CHDs.^6^ Individuals with CHDs often experience neurodevelopmental delays throughout childhood.^7^ Patients affected by CHDs also have an increased risk of acquired cardiovascular conditions and other health complications in later life.^6^^,^^8^^,^^9^ Continued improvements in early detection and a deeper understanding of CHD etiology have the potential to further enhance clinical management, support patient well-being, and inform strategies for prevention and personalized care.^9^

CHDs are often caused by complex genetic abnormalities that disrupt normal cardiac development,^10^ involving contributions from multiple genes, common and rare variants, and their interactions with environmental factors. Approximately 297 genes have been implicated in the development of CHDs.^11^^,^^12^ For example, *NKX2-5*, *GATA4*, *GATA5*, *GATA6*, and T-box transcriptional factors (*Tbx1*, *Tbx3*, *Tbx5*, and *Tbx20*) play pivotal roles in vertebrate cardiac development.^13^^,^^14^^,^^15^^,^^16^^,^^17^^,^^18^^,^^19^ However, despite significant progress in identifying CHD-susceptibility genes, the causes remain unknown for more than one-half of CHDs that occur.^6^^,^^20^ This knowledge gap may be attributed to multiple challenges facing genetic association analyses such that a single gene can cause a variety of defects and a single defect can have a variety of causes. CHDs encompass a large set of structural and functional deficits. For example, the level 3 classification of CHDs developed for the National Birth Defects Prevention Study (NBDPS) includes eight aggregated groups: anomalous pulmonary venous return, atrioventricular septal defect, complex, conotruncal, heterotaxy, left ventricular outflow tract obstruction, right ventricular outflow tract obstruction, and septal.^21^ These subtypes may be caused by a heterogeneous set of genetic variants that impact cardiac development.^22^ Existing statistical methods have commonly assumed homogeneous genetic effects across populations and families, and the high level of heterogeneity has limited the discovery of genetic causes of CHDs.^23^^,^^24^ Additionally, many existing analyses have focused on common genetic variants (e.g., minor allele frequencies >5%) in the population. Recent studies with sequencing data have showed that rare variants may contribute substantially to CHD development,^12^^,^^25^^,^^26^^,^^27^ but the identification of these variants usually requires large sample sizes. The application of advanced statistical methods that account for genetic heterogeneity and aggregate rare variants can increase the statistical power and provide a cost-effective way to uncover CHD-susceptibility genes.

Li et al. have recently proposed a family-based genetic random field (FGRF) method for detecting genetic variants with heterogeneous effects on complex traits.^28^ Similar to existing family-based association tests, FGRF utilizes within-family and between-family information separately or jointly to test an association. Extensive simulation studies showed that FGRF has statistical power comparable with that of existing methods when there is no genetic heterogeneity but can improve statistical power when there is genetic heterogeneity across families. In addition, FGRF is a gene- or region-based method based on the associations between genetic similarity and trait similarity. Therefore, it can aggregate information across all variants in a gene or region and is suitable for rare-variants association tests. The method was evaluated and discussed in detail elsewhere, and its application has identified rare genetic variants associated with alcohol dependence in the presence of disease heterogeneity.^28^

A genome-wide association study (GWAS) of obstructive CHDs was conducted using DNA collected from NBDPS participants. The initial analysis focused on testing individual common genetic variants for their association with the risk of obstructive CHDs.^29^ In the present study, we extended this work by applying the FGRF method to evaluate gene-level associations with the risk of obstructive CHDs. As we elaborate in the material and methods section below, we considered three versions of FGRF tests, which vary in their control of type I error and statistical power under scenarios of heterogeneous genetic effects. To investigate the biological relevance of the identified genes, we further conducted gene set enrichment analyses to elucidate the biological pathways involved.

## Material and methods

### Study population

Our study employed a family-based design comprising a mixture of infant-parental trios, infant-mother duos, infant-father duos, and infant-only families. It included 1,123 families with an infant affected by obstructive CHDs and 1,481 families with an unaffected infant. All participants were part of the NBDPS, one of the largest case-control studies of birth defects in the United States covering about 482,000 births annually.^30^^,^^31^ Our analysis included case infants who had one or more types of obstructive heart defects and were identified from population-based birth defect registries between 2003 and 2011 at nine participating sites: Arkansas, California, Georgia, Iowa, Massachusetts, New York, North Carolina, Texas, and Utah.^30^^,^^31^ Control infants did not have any known birth defects and were randomly selected from the same geographic area as the case infants using birth certificates or hospital records. Maternal exposures and other lifestyle factors that may influence embryogenesis were collected by research staff through a maternal computer-assisted telephone interview conducted 6–24 months after the estimated date of delivery, administered in English or Spanish. The CHD status of parents was determined by maternal responses to interview questions regarding the mother or father of the infant having a health problem at birth or birth defects diagnosed in childhood and review of the responses by a specialist in CHDs. Following the maternal interview, specimen collection kits that included cytobrushes (Cytology Brush Pack CYB-1, Medical Packaging Corp.) enclosed in paper-backed peel pouches that allowed air drying after collection were mailed to participating families. Participants returned the self-collected buccal cell specimens by mail in the provided paper envelopes.^32^^,^^33^ Buccal cell DNA specimens returned to five participating sites (Arkansas, California, Georgia, Iowa, and Texas) were included in the phase I GWAS, and DNA specimens from the other four participating sites (Massachusetts, New York, North Carolina, and Utah) were included in the phase II GWAS. Details of recruitment methods and data and specimen collection have been described in previous publications.^30^^,^^31^

### Genomic profiling and quality control

Buccal-cell-derived DNA specimens were genotyped through a two-phase GWAS using a case infant-mother-father trio and control infant-mother duo design.^29^ The genomic datasets and quality control were described elsewhere.^29^ In brief, each DNA specimen was genotyped at Arkansas Children’s Research Institute using Illumina’s Infinium Omni5Exome BeadChip which covered approximately 5 million single-nucleotide variants (SNVs). SNVs that had low call rates (<95%), extremely low minor allele frequencies (MAF < 0.0001) or deviated from Hardy-Weinberg equilibrium were removed. Samples with a genotype missing rate >5% were excluded. In addition, the concordance between clinically reported sex and genetic sex was verified using PLINK 1.9,^34^ and genetic relatedness between families was assessed using KING.^35^

### Genomic region selection

We referred to the UCSC Genome Browser (human genome assembly GRCh37/hg19) to define biologically meaningful candidate genomic regions for association tests. We used the software *bedtools* to merge redundant or overlapping genomic regions into a single consecutive region.^36^ A candidate region was defined as 7.5 kb upstream and downstream of each gene, reflecting a balance between capturing proximal *cis*-regulatory variants—which are enriched near transcription start sites and extend several kilobases beyond the gene body—and minimizing misassignment to neighboring genes,^37^^,^^38^ and a total of 24,270 autosomal regions were extracted for subsequent analysis. SNVs outside these boundaries were not included in the analysis. While linkage disequilibrium (LD) structure was not used to define regional boundaries, the FGRF method aggregates variant effects within each region through genetic similarity scores, which implicitly accounts for correlations among variants in LD.

### Region-based association tests by the FGRF method

We applied an FGRF method to test the association between each candidate region and CHD risk. The theoretical details of this method are provided elsewhere.^28^ In brief, the FGRF method uses spatial statistical modeling to integrate the effect of multiple genetic variants within the same region. When there is a gene-phenotype association, individuals with similar genotypes tend to exhibit similar phenotypes. To further leverage family-based samples, the FGRF method utilizes within-family and between-family information separately or jointly to test for an association. Phenotypes from both infants and parents were included when calculating similarity scores, and genetic similarity was then used to model shared genomic patterns within and across trios. This framework allows the method to borrow strength from genetically similar individuals and families, thereby improving power for detecting associations. The method is functionally agnostic and does not incorporate variant-level functional weighting. The within-family test is robust to potential confounding due to maternal exposures and population stratification, while the between-family test is not. In this study, we considered two versions of tests by the FGRF method with varying performance due to different scenarios of heterogeneous genetic effects. First, we conducted an overall test of the FGRF (FGRF-O) that utilizes both within-family and between-family information. FGRF-O is expected to maximize the statistical power to identify genes without heterogeneous effects in the population. Maternal exposures, including age at delivery, body mass index (BMI), race/ethnicity, total household income, education, periconceptional folic acid supplement use, and the top ten genomic principal components derived from individual genotype data using a kinship-aware principal-component analysis approach, PC-AiR,^39^ were adjusted for in the analysis. Second, we conducted a Fisher’s combined probability test of the FGRF (FGRF-F) that separately considered within-family and between-family information. FGRF-F is expected to have robust statistical power to identify genes with heterogeneous effects across families. FGRF-O uses all samples jointly in a single model and tends to be most powerful when genetic effects are relatively homogeneous across families. In contrast, FGRF-F combines separate within-family and between-family tests, making it more robust and often more powerful when genetic effects vary across families. Restricting analyses to obstructive CHD reduces phenotypic heterogeneity across families, while the FGRF method accounts for the residual genetic heterogeneity that remains within this subtype.

Within each region, we defined common and rare variants based on an MAF threshold of 0.05 and conducted separate tests for common variants, rare variants, and all variants. We performed these tests using samples from phase I and phase II independently, then combined the results from both phases using Fisher’s combined probability test. Because each approach (FGRF-O and FGRF-F) was used independently to evaluate regional associations, statistical significance was assessed using a Bonferroni correction that adjusts the threshold based on the total number of regions tested. Recognizing the conservative nature of this correction, we also identified regions with “suggestive” evidence of association (*p* values <10^−5^) in any of these tests, providing a more inclusive set of potentially significant results.

### Gene set enrichment analysis

To provide additional insights on the potential involvement of the identified genes in CHD development, we conducted gene set enrichment analyses to investigate whether the associated genes are overrepresented in any biological functions. We performed hypergeometric tests against gene sets from the GWAS Catalog, a comprehensive database of published genome-wide association studies,^40^ using the GENE2FUNC function within Functional Mapping and Annotation of Genome-Wide Association Studies platform.^41^ Genes with *p* values less than 0.05 were designated as the prioritized gene set. Because only a limited number of genes met a more stringent multiple testing threshold, using a nominal threshold helps retain sufficient power for enrichment analysis while allowing inclusion of moderately associated genes that may collectively reflect underlying biological pathways.^42^ This approach has been widely used in pathway analyses.^43^^,^^44^^,^^45^ The background gene set, which serves as the reference group, comprised 19,283 protein-coding genes. The GWAS Catalog served as the foundation for FUMA to generate gene sets associated with specific traits or diseases.^41^ We conducted hypergeometric tests for each gene set from the GWAS Catalog and applied multiple testing adjustment using the Benjamini-Hochberg method. Gene sets were reported if they had an adjusted *p* value less than 0.05 and at least one overlapping gene with the set of prioritized genes.

We additionally performed gene set enrichment analysis^46^ to assess whether cardiac-related gene sets were enriched among our list of genes, aiming to elucidate the biological roles of genes with small *p* values. Specifically, we extracted all gene sets from Gene Ontology that contained the terms “heart” or “cardiac” in their ontology term. These cardiac-specific gene sets were evaluated for enrichment against all genes in our study, ranked by their respective *p* values. Gene sets were reported if they had a nominal *p* value less than 0.05.

### Ethics statement

This study received approval from the Institutional Review Board at Indiana University Bloomington and University of Arkansas for Medical Sciences. Additionally, the NBDPS protocol was approved by the Institutional Review Board at the Centers for Disease Control and Prevention and all NBDPS centers. All study participants provided informed consent.

## Results

### Study population

Following removal of DNA specimens from 18 families due to presence of sex discrepancies or correlated families, the full study population comprised 5,665 individuals, including 1,166 individuals with obstructive CHDs (1,123 affected infants and 43 affected parents) and 4,499 control individuals, drawn from 2,604 families. The final analytical dataset included 708 parent-infant trios, 1,539 mother-infant duos, 106 father-infant duos, and 251 families with only an infant (Table 1). The phase I GWAS had 3,557 individuals from 662 case families and 969 control families, while the phase II GWAS had 2,108 individuals from 461 case families and 512 control families. The comparison of maternal characteristics and lifestyle factors between case and control families is presented in Table 2 for phase I and phase II. There was a significant difference in the distribution of race and ethnicity between case and control families in both phase I and phase II. Most mothers were non-Hispanic White (59.2% in phase I and 75.5% in phase II). Compared with mothers in control families, mothers in case families were more likely to be non-Hispanic White (65.3% vs. 57.4% in phase I; 79.4% vs. 72.1% in phase II) and less likely to be Hispanic (24.4% vs. 30.5% in phase I; 10.8% vs. 12.9% in phase II). The average maternal age at delivery was 27.3 years in phase I and 29.0 years in phase II. The maternal BMI was higher among case mothers than control mothers in phase II, and the difference was statistically significant (*p* = 0.005), while no significant difference was observed in phase I (*p* = 0.392). Case families had overall higher income levels than control families in phase I (*p* = 0.047), but no significant difference was observed in phase II. In phase I, case mothers tended to have higher education than control mothers (*p* = 0.029), but this difference was not statistically significant in phase II. In phase I, more case mothers reported using folic acid supplements compared to control mothers (*p* = 0.033), whereas in phase II, the usage of folic acid supplements was comparable between the two groups (*p* = 0.92). Additionally, the rates of maternal smoking and alcohol consumption did not differ significantly in either phase.

**Table 1:** Summary of family relationship groups in phase I and phase II genome-wide association study cohorts, the National Birth Defects Prevention Study, 2003–2011

	Phase I	Phase II	Total
Parent-infant trios	436	272	708
Mother-infant duos	984	555	1,539
Father-infant duos	70	36	106
Infant only	141	110	251

**Table 2:** Maternal characteristics of children born with congenital heart defects in the phase I and phase II genome-wide association study cohort, the National Birth Defects Prevention Study, 2003–2011

	Phase I				Phase II			
	Total (*N* = 1,631)	Case families (*n* = 662)	Control families (*n* = 969)	*p*value^a^	Total (*N* = 973)	Case families (*n* = 461)	Control families (*n* = 512)	*p*value^a^
Infant sex				0.141				0.672
Male	835 (51.2%)	354 (53.5%)	481 (49.6%)		526 (54.1%)	253 (54.9%)	273 (53.3%)	
Female	796 (48.8%)	308 (46.5%)	488 (50.4%)		447 (45.9%)	208 (45.1%)	239 (46.7%)	
Maternal age at delivery				0.007				0.421
Mean (SD)	27.28 (5.75)	27.75 (5.71)	26.96 (5.76)		28.98 (5.84)	29.14 (5.68)	28.84 (5.98)	
Maternal body mass index				0.392				0.005
Mean (SD)	26.49 (6.63)	26.66 (6.72)	26.37 (6.56)		25.38 (5.89)	25.95 (6.34)	24.87 (5.42)	
Maternal race/ethnicity				0.003				0.019
Non-Hispanic White	965 (59.2%)	409 (65.3%)	556 (57.4%)		735 (75.5%)	366 (79.4%)	369 (72.1%)	
Hispanic	449 (27.5%)	153 (24.4%)	296 (30.5%)		116 (11.9%)	50 (10.8%)	66 (12.9%)	
Other	217 (13.3%)	100 (16.0%)	117 (12.1%)		122 (12.5%)	45 (9.8%)	77 (15.0%)	
Total household income (US$)				0.047				0.981
<10,000	298 (18.3%)	100 (15.1%)	198 (20.4%)		112 (11.5%)	53 (11.5%)	59 (11.5%)	
10,000–20,000	221 (13.5%)	91 (13.7%)	130 (13.4%)		100 (10.3%)	44 (9.5%)	56 (10.9%)	
20,000–30,000	249 (15.3%)	95 (14.4%)	154 (15.9%)		104 (10.7%)	49 (10.6%)	55 (10.7%)	
30,000–40,000	183 (11.2%)	77 (11.6%)	106 (10.9%)		88 (9.0%)	44 (9.5%)	44 (8.6%)	
40,000–50,000	129 (7.9%)	60 (9.1%)	69 (7.1%)		85 (8.7%)	41 (8.9%)	44 (8.6%)	
>50,000	462 (28.3%)	204 (30.8%)	258 (26.6%)		447 (45.9%)	213 (46.2%)	234 (45.7%)	
Unknown	89 (5.5%)	35 (5.3%)	54 (5.6%)		37 (3.8%)	17 (3.7%)	20 (3.9%)	
Maternal education				0.029				0.638
0–8 years	99 (6.1%)	37 (5.6%)	62 (6.4%)		31 (3.2%)	17 (3.7%)	14 (2.7%)	
9–11 years	181 (11.1%)	61 (9.2%)	120 (12.4%)		65 (6.7%)	28 (6.1%)	37 (7.2%)	
12 years	433 (26.6%)	161 (24.4%)	272 (28.1%)		208 (21.4%)	104 (22.6%)	104 (20.3%)	
13–15 years	475 (29.2%)	201 (30.5%)	274 (28.3%)		253 (26.0%)	123 (26.7%)	130 (25.4%)	
≥16 years	441 (27.1%)	200 (30.3%)	241 (24.9%)		416 (42.8%)	189 (41.0%)	227 (44.3%)	
Periconceptional maternal cigarette smoking^b^				0.176				0.098
Yes	346 (21.2%)	152 (23.0%)	194 (20.0%)		165 (17.0%)	68 (14.8%)	97 (18.9%)	
No	1,284 (78.8%)	510 (77.0%)	774 (70.0%)		808 (83.0%)	393 (85.2%)	415 (81.1%)	
Periconceptional maternal alcohol use				0.394				0.794
Yes	592 (36.5%)	249 (37.8%)	343 (35.6%)		378 (39.0%)	176 (38.4%)	202 (39.5%)	
No	1,031 (63.5%)	410 (62.2%)	621 (64.4%)		592 (61.0%)	282 (61.6%)	310 (60.5%)	
Periconceptional folic acid supplement use				0.033				0.917
Yes	888 (54.4%)	382 (57.7%)	506 (52.2%)		619 (63.6%)	292 (63.3%)	327 (63.9%)	
No	743 (45.6%)	280 (42.3%)	463 (47.8%)		354 (36.4%)	169 (36.7%)	185 (36.1%)	

a Based on Pearson’s *χ*^2^ test for categorical variables and two-sample *t* test for continuous variables.

b This does not include marijuana, cigars, e-cigarettes, or vaping.

### Region-based association test by FGRF-O and FGRF-F

**Figure 1:**
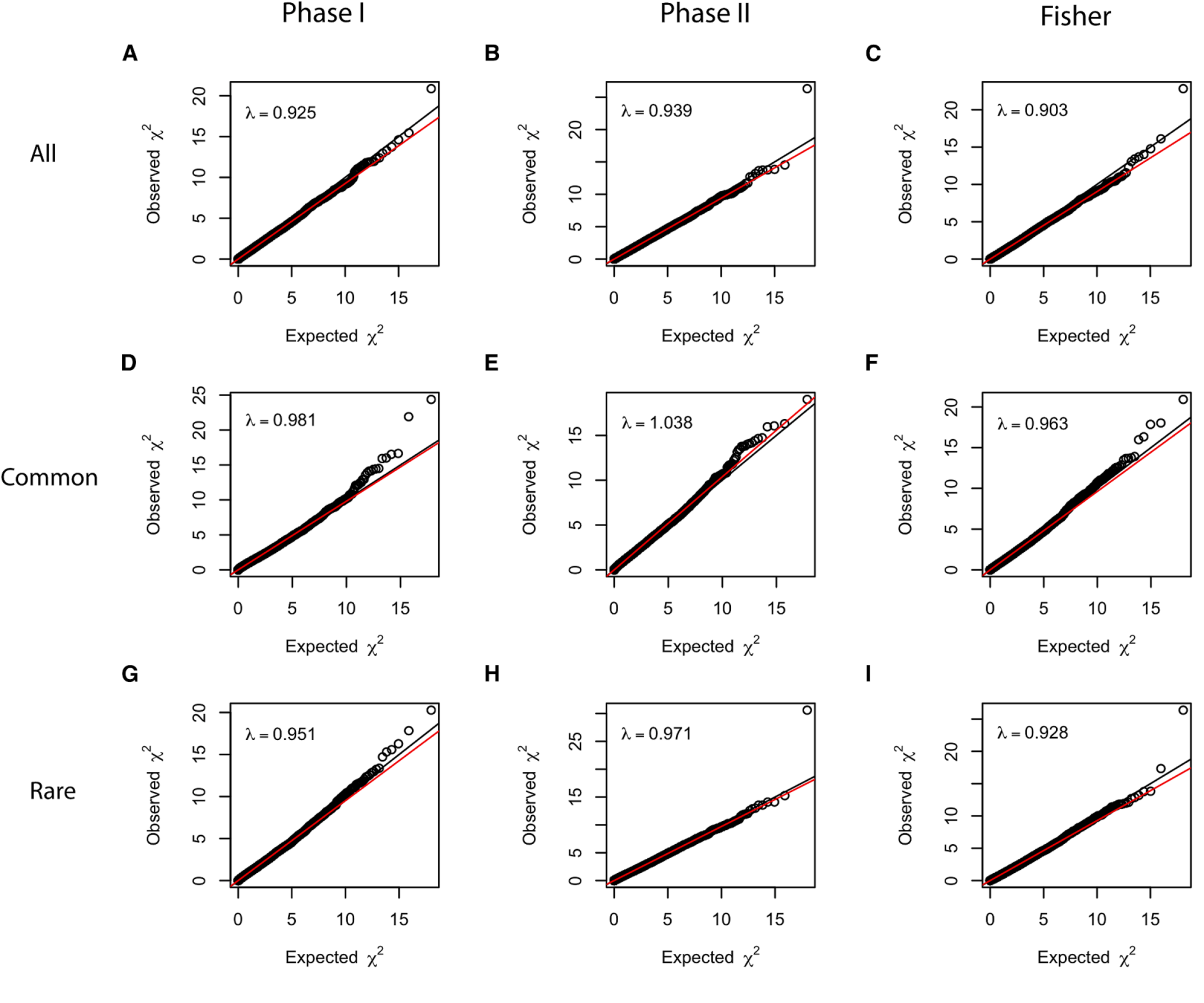
Quantile-quantile plots by applying the overall test of the family-based genetic random field to the genome-wide association study samples from the National Birth Defects Prevention Study, 2003–2011 (A) All-variants analysis in phase I. (B) All-variants analysis in phase II. (C) All-variants analysis in both phases using Fisher’s combinational test. (D) Common-variants analysis in phase I. (E) Common-variants analysis in phase II. (F) Common-variants analysis in both phases using Fisher’s combinational test. (G) Rare-variants analysis in phase I. (H) Rare-variants analysis in phase II. (I) Rare-variants analysis in both phases using Fisher’s combinational test.

**Figure 2:**
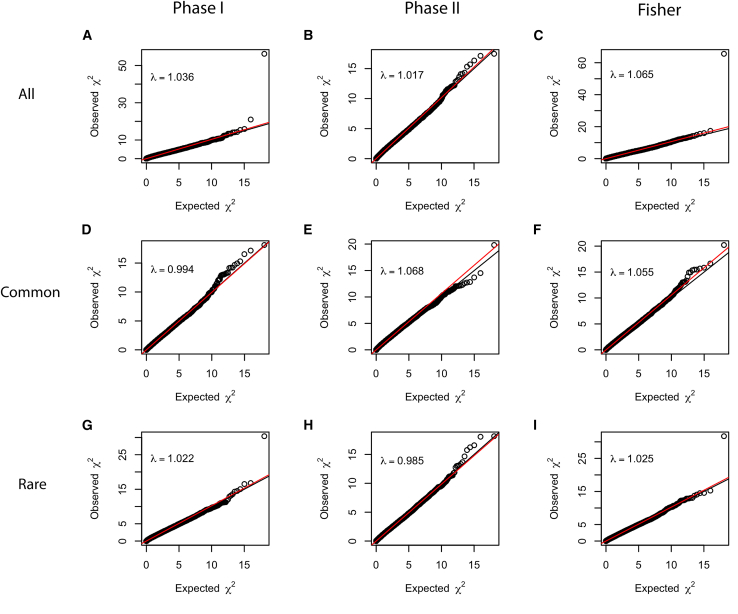
Quantile-quantile plots by applying Fisher’s combined probability test of the family-based genetic random field to the genome-wide association study samples from the National Birth Defects Prevention Study, 2003–2011 (A) All-variants analysis in phase I. (B) All-variants analysis in phase II. (C) All-variants analysis in both phases using Fisher’s combinational test. (D) Common-variants analysis in phase I. (E) Common-variants analysis in phase II. (F) Common-variants analysis in both phases using Fisher’s combinational test. (G) Rare-variants analysis in phase I. (H) Rare-variants analysis in phase II. (I) Rare-variants analysis in both phases using Fisher’s combinational test.

**Figure 3:**
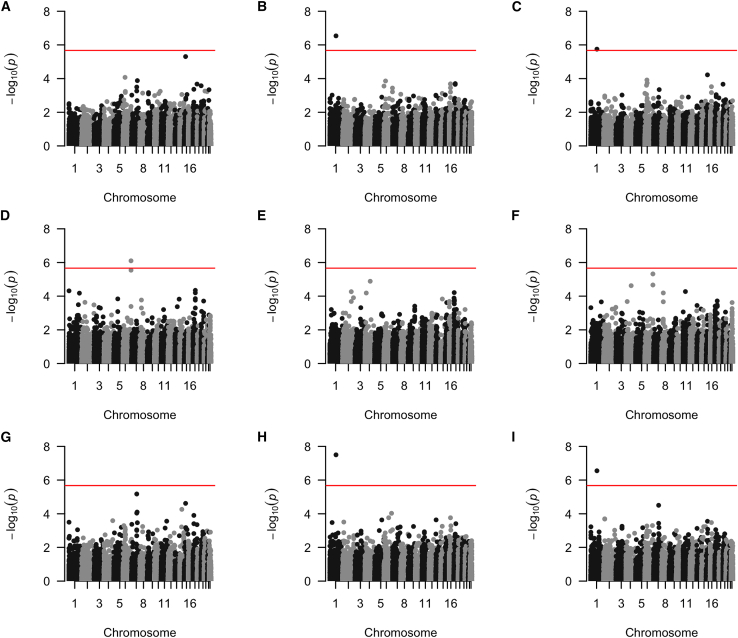
Manhattan plots by applying the overall test of the family-based genetic random field to the genome-wide association study samples from the National Birth Defects Prevention Study, 2003–2011 (A) All-variants analysis in phase I. (B) All-variants analysis in phase II. (C) All-variants analysis in both phases using Fisher’s combinational test. (D) Common-variants analysis in phase I. (E) Common-variants analysis in phase II. (F) Common-variants analysis in both phases using Fisher’s combinational test. (G) Rare-variants analysis in phase I. (H) Rare-variants analysis in phase II. (I) Rare-variants analysis in both phases using Fisher’s combinational test. The red line indicates the Bonferroni-corrected significance level.

**Figure 4:**
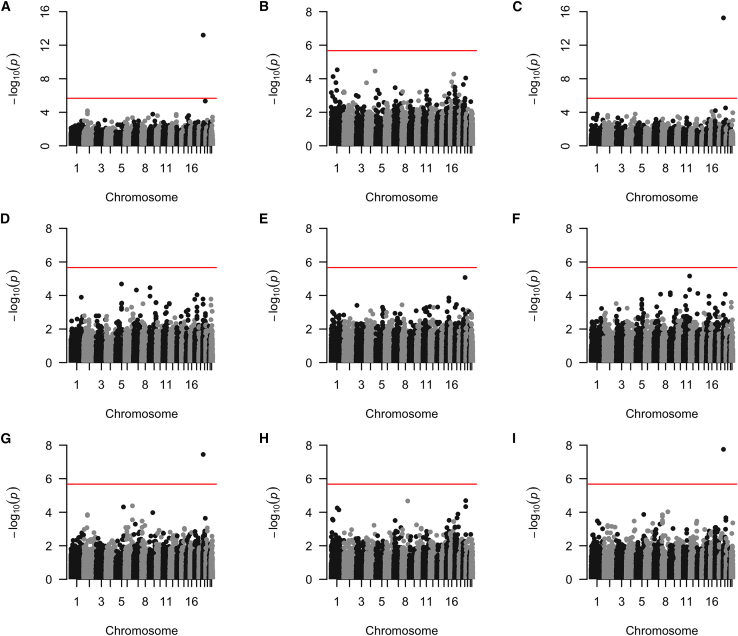
Manhattan plots by applying the Fisher’s combined probability test of the family-based genetic random field to the genome-wide association study samples from the National Birth Defects Prevention Study, 2003–2011 (A) All-variants analysis in phase I. (B) All-variants analysis in phase II. (C) All-variants analysis in both phases using Fisher’s combinational test. (D) Common-variants analysis in phase I. (E) Common-variants analysis in phase II. (F) Common-variants analysis in both phases using Fisher’s combinational test. (G) Rare-variants analysis in phase I. (H) Rare-variants analysis in phase II. (I) Rare-variants analysis in both phases using Fisher’s combinational test. The red line indicates the Bonferroni-corrected significance level.

After genotype quality control, 3,263,047 SNVs remained in both study phases. For each genomic region, we examined common variants, rare variants, and all variants separately for their association with CHD risk using FGRF-F and FGRF-O. A total of 23,043 genomic regions were tested for common variants, 23,527 for rare variants, and 23,621 for all variants. Accordingly, the Bonferroni-corrected significance thresholds were 2.17 × 10^−6^, 2.13 × 10^−6^, and 2.12 × 10^−6^ for the common-, rare-, and all-variants analyses, respectively. The distribution of *p* values was evaluated against a uniform distribution using quantile-quantile plots, as shown in Figure 1 for FGRF-O and Figure 2 for FGRF-F. The genomic inflation factors ranged from 0.903 to 1.038 for FGRF-O tests and from 0.985 to 1.068 for FGRF-F tests, suggesting that type I errors were well controlled. The Manhattan plots for FGRF-O and FGRF-F are presented in Figures 3 and 4, respectively.

Three genes were found to be significantly associated with obstructive CHD risk after Bonferroni adjustment, with the *p* values for all analyses listed in Table 3. The genes *SERAC1* and *ZNF697* were identified by FGRF-O in common- and rare-variants analyses, respectively, with significant associations in one phase that failed to replicate in the other phase. The third gene, *SLC44A2*, was identified by FGRF-F in rare-variants and all-variants analyses, with significant results in phase I that were replicated in phase II (with at least nominal significance) and in the combined analysis. To elaborate, *SLC44A2* showed significant association with CHD in the rare-variants analysis, achieving a Bonferroni-corrected significance level in phase I (*p* = 3.58 × 10^−8^), a nominal significance level in phase II (*p* = 2.27 × 10^−2^), and a significant association after combining the phase I and phase II results using Fisher’s combined probability test (*p* = 1.79 × 10^−8^). Moreover, stronger associations in this gene were observed in our all-variants analysis (phase I *p* = 6.31 × 10^−14^, phase II *p* = 2.21 × 10^−4^, and Fisher’s combined probability tests *p* = 5.55 × 10^−16^). The relatively small *p* values in the rare-variants analysis suggest the potential cumulative effects of rare variants within this gene. Regional genomic annotation plots for the three significant gene-associated regions, including publicly available Hi-C chromatin interaction and ENCODE H3K27ac data, are shown in Figure S1. In addition to the Bonferroni significant findings, we identified an additional genomic region and nearby gene with false discovery rate (FDR) *q* values less than 0.05 (Table 4). Compared with Bonferroni correction, which strictly controls the family-wise error rate, FDR provides a less conservative approach for identifying potentially relevant associations while accounting for multiple testing.

**Table 3:** Genomic regions and nearby genes significantly associated with congenital heart defects after Bonferroni correction, the National Birth Defects Prevention Study, 2003–2011

	Chr	Region	Gene	*p* value									No. of SNVs in region	
				Common-variants analysis			Rare-variants analysis			All-variants analysis				
				Phase I	Phase II	Fisher	Phase I	Phase II	Fisher	Phase I	Phase II	Fisher	Common	Rare
FGRF-O	1	120161999–120190390	*ZNF697*	7.56 × 10^−1^	1.32 × 10^−1^	3.30 × 10^−1^	4.66 × 10^−1^	3.16 × 10^−8^	2.80 × 10^−7^	3.60 × 10^−1^	2.90 × 10^−7^	1.78 × 10^−6^	36	34
	6	158530535–158589312	*SERAC1*	7.92 × 10^−7^	3.76 × 10^−1^	4.78 × 10^−6^	4.80 × 10^−1^	8.29 × 10^−1^	7.64 × 10^−1^	2.00 × 10^−1^	>0.999	5.22 × 10^−1^	35	50
FGRF-F	19	10713120–10755235	*SLC44A2*	4.56 × 10^−1^	4.93 × 10^−1^	5.60 × 10^−1^	3.58 × 10^−8^	2.27 × 10^−2^	1.79 × 10^−8^	6.31 × 10^−14^	2.21 × 10^−4^	5.55 × 10^−16^	13	24

Chr, chromosome; SNV, single-nucleotide variant; FGRF-O, family-based genetic random field, overall test; FGRF-F, family-based genetic random field, Fisher’s combined probability test.

**Table 4:** Genomic region and nearby gene associated with congenital heart defects at false discovery rate *q* value <0.05, the National Birth Defects Prevention Study, 2003–2011

	Chr	Region	Gene	*p* value									No. of SNVs in region	
				Common-variants analysis			Rare-variants analysis			All-variants analysis				
				Phase I	Phase II	Fisher	Phase I	Phase II	Fisher	Phase I	Phase II	Fisher	Common	Rare
FGRF-O	6	158589378–158620376	*GTF2H5*	2.87 × 10^−6^^a^	5.26 × 10^−1^	2.17 × 10^−5^	6.65 × 10^−1^	3.82 × 10^−1^	6.02 × 10^−1^	2.99 × 10^−1^	5.79 × 10^−1^	4.77 × 10^−1^	38	34

Chr, chromosome; SNV, single-nucleotide variant; FGRF-O, family-based genetic random field, overall test.

a *q* value = 0.033.

Genomic regions and nearby genes with *p* values less than 10^−5^ in any analysis are additionally provided in Table S1. Among these suggestive findings, the gene *STT3A-AS1*, replicated across phases, produced consistently small *p* values in common-variants analysis of phase I (*p* = 2.99 × 10^−4^), phase II (*p* = 1.48 × 10^−3^), and Fisher’s combined probability test (*p* = 6.93 × 10^−6^), suggesting a moderately significant association, even though it did not reach the Bonferroni-corrected significance threshold. In addition, except for the genes in Table 3, the remaining genes presented here typically exhibited *p* values near the Bonferroni-adjusted significance level in either common- or rare-variants analysis of one phase, without replication in the other phase. For example, the gene *EPO* identified by FGRF-O had a *p* value of 6.70 × 10^−6^ in rare-variants analysis of phase I samples, just above the Bonferroni-corrected significance threshold of 2.13 × 10^−6^. However, *EPO* was not associated with obstructive CHDs in phase II. The small *p* value obtained in Fisher’s combined probability test of rare-variants analysis was predominantly influenced by the result in phase I. Similar results were observed for this gene in the all-variants analysis.

### Gene set enrichment analysis

**Figure 5:**
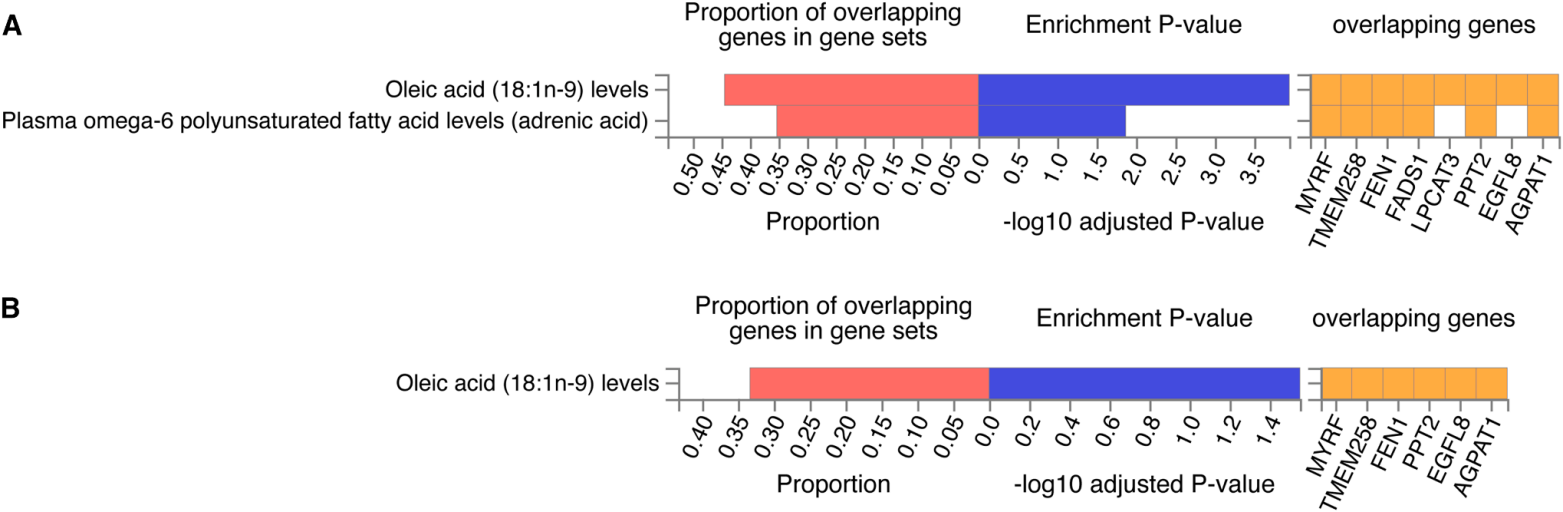
Overrepresentation of gene sets identified by a family-based genetic random field method in reported gene sets from the GWAS Catalog (A) Overrepresentation of genes identified in common-variants analysis by FGRF-O. (B) Overrepresentation of genes identified in all-variants analysis by FGRF-O. National Birth Defects Prevention Study, 2003–2011.

We examined the overrepresentation of genes with *p* < 0.05 across various biological functions. Figure 5 displays the gene sets from the GWAS Catalog in which our prioritized genes were significantly overrepresented. Among genes with *p* < 0.05 in the common variants analysis by FGRF-O, two gene sets were identified with an FDR of 0.05 after Benjamini-Hochberg adjustment: genes related to oleic acid levels and plasma omega-6 polyunsaturated fatty acid levels. The gene set related to oleic acid levels also had an adjusted *p* value of less than 0.05 when the genes prioritized in all variants analysis by FGRF-O were tested for overrepresentation. No significant gene sets were reported for genes prioritized in the rare variants analysis by FGRF-O or the all variants analysis by FGRF-F.

We also tested the overrepresentation of heart-related gene sets within our ranked genes, which were sorted according to their *p* values. From the Gene Ontology database, we extracted a total of 148 gene sets related to heart function for this analysis. Table 5 lists the gene sets that were significantly enriched at a nominal significance level of 0.05. For the ranked gene list derived analyses by FGRF-O, one gene set was significantly enriched in the analysis of common variants, rare variants, and all variants: genes associated with any process positively regulating the development of a cardiac muscle cell by influencing the rate, frequency, or extent of cell growth. These processes facilitate the progression of cells from initial formation to mature status. Five additional gene sets were significantly enriched in the common-variants analysis exclusively, two of which are associated with the regulation of growth and development in cardiac cells. For the ranked gene list derived from results by FGRF-F, one gene set was found to be significantly enriched in all-variants analysis: genes associated with any process negatively regulating cardiac muscle cell proliferation.

**Table 5:** Gene Ontology gene sets significantly enriched at nominal *p* value <0.05 in pre-ranked gene lists based on *p* values by different family-based genetic random field analyses, the National Birth Defects Prevention Study, 2003–2011

	Genetic variant type	Gene set	No. of genes in gene set	Enrichment score	Normalized enrichment score	*p* value
FGRF-O	common	GOBP physiological cardiac muscle hypertrophy	25	0.29	1.75	0.022
		GOBP regulation of cell growth involved in cardiac muscle cell development	16	0.36	1.72	0.034
		GOBP cardiac cell development	81	0.16	1.72	0.022
		GOBP membrane depolarization during cardiac muscle cell action potential	22	0.29	1.63	0.032
		GOBP cardiac muscle cell cardiac muscle cell adhesion	7	0.48	1.57	0.039
		GOBP positive regulation of cell growth involved in cardiac muscle cell development	6	0.52	1.54	0.041
	rare	GOBP positive regulation of cell growth involved in cardiac muscle cell development	7	0.53	1.77	0.022
	all	GOBP positive regulation of cell growth involved in cardiac muscle cell development	7	0.51	1.68	0.030
FGRF-F	all	GOBP negative regulation of cardiac muscle cell proliferation	17	0.36	1.83	0.016

FGRF-O, family-based genetic random field, overall test; FGRF-F, family-based genetic random field, Fisher’s combined probability test; GOBP, Gene Ontology Biological Process.

## Discussion

This study extended previous research on the identification of genetic risk factors for CHDs by applying FGRF to genotyping data from NBDPS participants. We assessed the association between a set of genomic regions and the risk of obstructive heart defects, and several genes showed an association with obstructive CHDs at a Bonferroni-corrected significance level. We hypothesize that these genes may contribute to obstructive CHD development with subtle to moderate effect sizes, operating via an intricate interaction mechanism with other variants. The findings are complementary to the genetic variants with larger magnitudes of effect estimates that have been identified by other methods.^29^^,^^47^ The *in silico* functional validation of our findings provides further insights into the biological mechanisms through which these genes may influence the susceptibility to obstructive CHDs.

Three genes reached Bonferroni-adjusted significance in our analyses; however, their levels of supporting evidence differ. Gene *SLC44A2* emerged as the most robust finding, showing a significant association with obstructive CHDs in rare-variants analysis using phase I samples and Fisher’s combined probability test. This association was replicated in phase II at the nominal significance level, reinforcing the initial observation from phase I. When common and rare variants were analyzed jointly, the association became even more pronounced, yielding highly significant results in both phase I analysis and the combined analysis. The likelihood that genotyping errors appearing as “extremely rare variants” drove the main finding is low, as the top SNV reported in Rashkin et al.^29^ was also located in *SLC44A2* and had a reasonable MAF, which may have contributed to the signal observed in our study. In contrast, the other two genes showed phase-specific significance that was not replicated, suggesting that these findings should be interpreted with caution and may represent false positives due to random variation. However, as we discussed earlier, it is also worthwhile to note that causal genes, especially rare variants, are likely to have heterogeneous effects across families. Given that rare variants with strong effects may be exclusive to specific families or even unique to individual families, it is conceivable that such variants might go undetected. In addition, as the within-family component is robust to population stratification, the genes identified by FGRF-F are more likely than those identified by FGRF-O to have heterogeneous effects across subpopulations. Given the lack of replication, confidence in these associations is limited. Therefore, these genes should be considered preliminary and require independent validation before biological interpretation.

In our analyses, we have used reference genome GRCH37/hg19 to define gene regions to be consistent with the initial GWAS.^29^ The mapping of gene regions to the most recent GRCh38/hg38 can be done using UCSC LiftOver.^48^ To facilitate comparison with current genomic resources, we have provided the gene regions for top three genes (*ZNF697*, *SERAC1*, and *SLC44A2*) under assembly GRCH38/hg38 in Table S2. The regions of these genes are largely consistent between two reference genomes.

The *SLC44A2* gene was identified with replicable association across two phases. This gene, solute carrier family 44 member 2, is involved in choline transmembrane transporter activity and conversion from choline to betaine. Although observed association does not establish causality, prior studies have suggested that choline exposure during pregnancy affects the methylation of histone and DNA in the placenta and growing embryo.^29^^,^^49^ Choline also plays a regulatory role in the levels of *S*-adenosylhomocysteine and *S*-adenosylmethionine, changes in which have been associated with CHD.^50^^,^^51^ Further experimental investigation is needed to establish whether SLC44A2 has a causal role in CHD development. Additionally, the SNV rs2360743, a rare intronic variant in *SLC44A2*, reached genome-wide significance in a previous SNV-level association study of the same dataset, where it was reported as one of the top variants associated with obstructive heart defects.^29^ Based on this prior finding, it seems likely that this SNV may be a major contributor toward the observed gene-level associations we detected. It should be noted that the identified genes should be interpreted as gene-centered association signals rather than definitive causal genes. Because variants were assigned to genes based on genomic proximity, the observed associations may also reflect regulatory effects on nearby or more distal genes.

Genetic association studies have benefited greatly from the success of GWASs in identifying common variants associated with common diseases. However, the power of GWASs in detecting rare variants is limited due to the heterogeneous nature, low minor allele frequencies, and marginal effects of these variants. The FGRF method can effectively detect the combined effect of multiple genetic variants with marginal effects, especially in the case of rare variants. The family-based design makes it robust to population stratification. Moreover, FGRF leverages within- and between-family genetic similarities to assess the collective effect of each genomic region, making it particularly appropriate for association studies in scenarios where genetic heterogeneity exists. Therefore, FGRF can serve as a complementary method to a conventional GWAS for detecting the genetic effects of rare variants on human diseases.

While this analysis has yielded valuable insights, it is important to note a few limitations that could impact the interpretation and practical applications of our findings. First, FGRF is a gene-based test that assesses the cumulative effect of multiple genetic variants within a gene, where associations are evaluated at the gene level rather than the individual-variant level. Like other gene-based methods, it cannot pinpoint causal variants or estimate their effect size for individual variants. Consequently, significant gene-level associations may reflect the cumulative effects of multiple variants or may be driven primarily by one or a few variants within the tested region. For information purposes, the minor allele frequencies of variants within the identified genes and approximate effect estimates from single-variant analyses are provided in Table S3. Further research is needed to identify and quantify the effects of causal variants within the gene of interest. Second, the phenotype of parents in our study was based on self-reported descriptions of birth defects, which may not provide an accurate representation of obstructive CHD. This could have some bearing on the performance of FGRF and the reliability of the results obtained. Additionally, the maternal interview was conducted 6–24 months after the estimated date of delivery, and parental CHD status was assessed solely through maternal responses. The wide interview window may introduce recall bias, while reliance on maternal report alone to ascertain paternal CHD status may lead to inaccuracies in phenotype classification, potentially affecting the performance of FGRF and the reliability of our results. Third, the samples used in the two study phases were drawn from non-overlapping centers and exhibited differences in demographic characteristics, which could affect the comparability of results across phases. While the statistically significant difference in maternal age between cases and controls in phase I (mean age 27.8 vs. 27.0 years; *p* = 0.007) was unlikely to be of major clinical significance, broader demographic heterogeneity between phases may contribute to reduced replication of findings, especially for rare-variants analyses that are sensitive to sample composition. Fourth, gene-based tests may have reduced power when there are only a few susceptibility loci with significant effects within the region, particularly when a large number of genetic variants are being tested. Finally, future studies incorporating targeted sequencing and functional genomic profiling (e.g., ATAC-seq^52^) will be valuable for identifying the causal variants and validating their regulatory roles.^53^

In conclusion, by applying the FGRF-O and FGRF-F approaches, we identified gene *SLC44A2* to have replicable associations with obstructive CHD risk based on analysis of 2,604 families from the NBDPS. Although two additional genes, *ZNF697* and *SERAC1*, showed significant associations that were not replicated, they are candidate genes that can be evaluated in other cohorts. Through functional characterization of the top candidate genes, we found evidence suggesting their involvement in the positive regulation of cell growth during cardiac muscle cell development. Our study underscores the genetic contribution to obstructive CHD risk at the gene level and offers potential implications in the biological mechanism through which these genes may influence disease susceptibility. The findings highlight candidate genes that future biological experiments could investigate in greater depth.

## Data and code availability

The genetic data from NBDPS is not publicly available, due to the restriction of GWAS data and protected health information. Access to the data can be requested, and the procedure is outlined at the following link: https://www.cdc.gov/birth-defects/php/bd-steps-nbdps-data/?CDC_AAref_Val=https://www.cdc.gov/ncbddd/birthdefects/nbdps-public-access-procedures.html. The code used in this paper can be located at the following GitHub repository: https://github.com/manyan-huang/FGRF-CHD.git.

## Acknowledgments

The authors wish to thank study subjects for their participation in the National Birth Defects Prevention Study (NBDPS) and are appreciative of the contributions made by NBDPS and BD-STEPS scientists and staff. This project was supported through Centers for Disease Control and Prevention (10.13039/100000030CDC) cooperative agreements under 10.13039/100006131PA #96043, PA #02081, FOA #DD09-001, FOA #DD13-003, NOFO #DD18-001, and NOFO #DD23-001 to the Centers for Birth Defects Research and Prevention participating in the National Birth Defects Prevention Study (NBDPS) and/or the Birth Defects Study To Evaluate Pregnancy Exposures (BD-STEPS) (AR U01DD001306, CA U01DD001302, MA U01DD001300, NC U01DD001308, NY U01DD001304, and DD001227; and TX U01DD001309), and the 10.13039/100000050National Heart, Lung, and Blood Institute under award numbers K01HL140333, R56HL164477, and R01HL171194. The content is solely the responsibility of the authors and does not necessarily represent the official views of the 10.13039/100000002National Institutes of Health or 10.13039/100000030CDC.

## Author contributions

M.H. and M.L. conceived and designed the study, performed the analysis, and wrote the manuscript; N.L. and J.S.W. contributed to the analysis tools; and S.M.W., J.S.W., B.D.G., W.N.N., M.W., M.M.H., L.B., A.J.A., O.O.O., M.M.J., L.M.A., A.O., P.A.R., and G.S. provided critical comments. All authors read and approved the manuscript.

## Declaration of interests

The authors declare no competing interests.

## Supplemental information

Document S1. Figure S1 and Tables S1–S3

Document S2. Article plus supplemental information

## References

[1] Su W., Zhu P., Wang R., Wu Q., Wang M., Zhang X., Mei L., Tang J., Kumar M., Wang X. (2017). Congenital heart diseases and their association with the variant distribution features on susceptibility genes. Clin. Genet..

[2] Khoshnood B., Lelong N., Houyel L., Thieulin A.C., Jouannic J.M., Magnier S., Delezoide A.L., Magny J.F., Rambaud C., Bonnet D. (2012). Prevalence, timing of diagnosis and mortality of newborns with congenital heart defects: a population-based study. Heart.

[3] Pierpont M.E., Brueckner M., Chung W.K., Garg V., Lacro R.V., McGuire A.L., Mital S., Priest J.R., Pu W.T., Roberts A. (2018). Genetic Basis for Congenital Heart Disease: Revisited: A Scientific Statement From the American Heart Association. Circulation.

[4] Houyel L., Meilhac S.M. (2021). Heart Development and Congenital Structural Heart Defects. Annu. Rev. Genom. Hum. Genet..

[5] Rosamond W., Flegal K., Friday G., Furie K., Go A., Greenlund K., Haase N., Ho M., Howard V., Kissela B. (2007). Heart disease and stroke statistics--2007 update: a report from the American Heart Association Statistics Committee and Stroke Statistics Subcommittee. Circulation.

[6] Morton S.U., Quiat D., Seidman J.G., Seidman C.E. (2022). Genomic frontiers in congenital heart disease. Nat. Rev. Cardiol..

[7] Patt E., Singhania A., Roberts A.E., Morton S.U. (2023). The Genetics of Neurodevelopment in Congenital Heart Disease. Can. J. Cardiol..

[8] Nicoll R. (2018). Environmental Contaminants and Congenital Heart Defects: A Re-Evaluation of the Evidence. Int. J. Environ. Res. Publ. Health.

[9] Liu A., Diller G.P., Moons P., Daniels C.J., Jenkins K.J., Marelli A. (2023). Changing epidemiology of congenital heart disease: effect on outcomes and quality of care in adults. Nat. Rev. Cardiol..

[10] Nees S.N., Chung W.K. (2020). Genetic Basis of Human Congenital Heart Disease. Cold Spring Harbor Perspect. Biol..

[11] Williams K., Carson J., Lo C. (2019). Genetics of Congenital Heart Disease. Biomolecules.

[12] Sierant M.C., Jin S.C., Bilguvar K., Morton S.U., Dong W., Jiang W., Lu Z., Li B., López-Giráldez F., Tikhonova I. (2025). Genomic analysis of 11,555 probands identifies 60 dominant congenital heart disease genes. Proc. Natl. Acad. Sci. USA.

[13] Granados-Riveron J.T., Pope M., Bu’lock F.A., Thornborough C., Eason J., Setchfield K., Ketley A., Kirk E.P., Fatkin D., Feneley M.P. (2012). Combined mutation screening of NKX2-5, GATA4, and TBX5 in congenital heart disease: multiple heterozygosity and novel mutations. Congenit. Heart Dis..

[14] Greulich F., Rudat C., Kispert A. (2011). Mechanisms of T-box gene function in the developing heart. Cardiovasc. Res..

[15] He A., Kong S.W., Ma Q., Pu W.T. (2011). Co-occupancy by multiple cardiac transcription factors identifies transcriptional enhancers active in heart. Proc. Natl. Acad. Sci. USA.

[16] Harvey R.P., Lai D., Elliott D., Biben C., Solloway M., Prall O., Stennard F., Schindeler A., Groves N., Lavulo L. (2002). Homeodomain factor Nkx2-5 in heart development and disease. Cold Spring Harbor Symp. Quant. Biol..

[17] Peterkin T., Gibson A., Loose M., Patient R. (2005). The roles of GATA-4, -5 and -6 in vertebrate heart development. Semin. Cell Dev. Biol..

[18] McCulley D.J., Black B.L. (2012). Transcription factor pathways and congenital heart disease. Curr. Top. Dev. Biol..

[19] Prendiville T., Jay P.Y., Pu W.T. (2014). Insights into the genetic structure of congenital heart disease from human and murine studies on monogenic disorders. Cold Spring Harb. Perspect. Med..

[20] Szot J.O., Cuny H., Blue G.M., Humphreys D.T., Ip E., Harrison K., Sholler G.F., Giannoulatou E., Leo P., Duncan E.L. (2018). A Screening Approach to Identify Clinically Actionable Variants Causing Congenital Heart Disease in Exome Data. Circ. Genom. Precis. Med..

[21] Botto L.D., Lin A.E., Riehle-Colarusso T., Malik S., Correa A., National Birth Defects Prevention Study (2007). Seeking causes: Classifying and evaluating congenital heart defects in etiologic studies. Birth Defects Res. A Clin. Mol. Teratol..

[22] Cowan J.R., Ware S.M. (2015). Genetics and genetic testing in congenital heart disease. Clin. Perinatol..

[23] Yuan S., Zaidi S., Brueckner M. (2013). Congenital heart disease: emerging themes linking genetics and development. Curr. Opin. Genet. Dev..

[24] Duncan A.R., Khokha M.K. (2016). Xenopus as a model organism for birth defects-Congenital heart disease and heterotaxy. Semin. Cell Dev. Biol..

[25] Jin S.C., Homsy J., Zaidi S., Lu Q., Morton S., DePalma S.R., Zeng X., Qi H., Chang W., Sierant M.C. (2017). Contribution of rare inherited and de novo variants in 2,871 congenital heart disease probands. Nat. Genet..

[26] Watkins W.S., Hernandez E.J., Wesolowski S., Bisgrove B.W., Sunderland R.T., Lin E., Lemmon G., Demarest B.L., Miller T.A., Bernstein D. (2019). De novo and recessive forms of congenital heart disease have distinct genetic and phenotypic landscapes. Nat. Commun..

[27] Yu M., Aguirre M., Jia M., Gjoni K., Cordova-Palomera A., Munger C., Amgalan D., Rosa Ma X., Pereira A., Tcheandjieu C. (2023). Oligogenic Architecture of Rare Noncoding Variants Distinguishes 4 Congenital Heart Disease Phenotypes. Circ. Genom. Precis. Med..

[28] Li M., He Z., Tong X., Witte J.S., Lu Q. (2018). Detecting Rare Mutations with Heterogeneous Effects Using a Family-Based Genetic Random Field Method. Genetics.

[29] Rashkin S.R., Cleves M., Shaw G.M., Nembhard W.N., Nestoridi E., Jenkins M.M., Romitti P.A., Lou X.Y., Browne M.L., Mitchell L.E. (2022). A genome-wide association study of obstructive heart defects among participants in the National Birth Defects Prevention Study. Am. J. Med. Genet..

[30] Yoon P.W., Rasmussen S.A., Lynberg M.C., Moore C.A., Anderka M., Carmichael S.L., Costa P., Druschel C., Hobbs C.A., Romitti P.A. (2001). The National Birth Defects Prevention Study. Publ. Health Rep..

[31] Reefhuis J., Gilboa S.M., Anderka M., Browne M.L., Feldkamp M.L., Hobbs C.A., Jenkins M.M., Langlois P.H., Newsome K.B., Olshan A.F. (2015). The National Birth Defects Prevention Study: A review of the methods. Birth Defects Res. A Clin. Mol. Teratol..

[32] Gallagher M.L., Sturchio C., Smith A., Koontz D., Jenkins M.M., Honein M.A., Rasmussen S.A. (2011). Evaluation of mailed pediatric buccal cytobrushes for use in a case-control study of birth defects. Birth Defects Res. A Clin. Mol. Teratol..

[33] Rasmussen S.A., Lammer E.J., Shaw G.M., Finnell R.H., McGehee R.E., Gallagher M., Romitti P.A., Murray J.C., National Birth Defects Prevention Study (2002). Integration of DNA sample collection into a multi-site birth defects case-control study. Teratology.

[34] Chang C.C., Chow C.C., Tellier L.C., Vattikuti S., Purcell S.M., Lee J.J. (2015). Second-generation PLINK: rising to the challenge of larger and richer datasets. GigaScience.

[35] Manichaikul A., Mychaleckyj J.C., Rich S.S., Daly K., Sale M., Chen W.-M. (2010). Robust relationship inference in genome-wide association studies. Bioinformatics.

[36] Quinlan A.R., Hall I.M. (2010). BEDTools: a flexible suite of utilities for comparing genomic features. Bioinformatics.

[37] Pickrell J.K., Marioni J.C., Pai A.A., Degner J.F., Engelhardt B.E., Nkadori E., Veyrieras J.B., Stephens M., Gilad Y., Pritchard J.K. (2010). Understanding mechanisms underlying human gene expression variation with RNA sequencing. Nature.

[38] Petersen A., Alvarez C., DeClaire S., Tintle N.L. (2013). Assessing methods for assigning SNPs to genes in gene-based tests of association using common variants. PLoS One.

[39] Conomos M.P., Miller M.B., Thornton T.A. (2015). Robust inference of population structure for ancestry prediction and correction of stratification in the presence of relatedness. Genet. Epidemiol..

[40] Welter D., MacArthur J., Morales J., Burdett T., Hall P., Junkins H., Klemm A., Flicek P., Manolio T., Hindorff L., Parkinson H. (2014). The NHGRI GWAS Catalog, a curated resource of SNP-trait associations. Nucleic Acids Res..

[41] Watanabe K., Taskesen E., van Bochoven A., Posthuma D. (2017). Functional mapping and annotation of genetic associations with FUMA. Nat. Commun..

[42] Farber C.R. (2013). Systems-Level Analysis of Genome-Wide Association Data. G3 (Bethesda).

[43] White M.J., Yaspan B.L., Veatch O.J., Goddard P., Risse-Adams O.S., Contreras M.G. (2019). Strategies for Pathway Analysis Using GWAS and WGS Data. Curr. Protoc. Hum. Genet..

[44] O’Dushlaine C., Kenny E., Heron E.A., Segurado R., Gill M., Morris D.W., Corvin A. (2009). The SNP ratio test: pathway analysis of genome-wide association datasets. Bioinformatics.

[45] Weng L., Macciardi F., Subramanian A., Guffanti G., Potkin S.G., Yu Z., Xie X. (2011). SNP-based pathway enrichment analysis for genome-wide association studies. BMC Bioinf..

[46] Subramanian A., Tamayo P., Mootha V.K., Mukherjee S., Ebert B.L., Gillette M.A., Paulovich A., Pomeroy S.L., Golub T.R., Lander E.S., Mesirov J.P. (2005). Gene set enrichment analysis: a knowledge-based approach for interpreting genome-wide expression profiles. Proc. Natl. Acad. Sci. USA.

[47] Cordell H.J., Bentham J., Topf A., Zelenika D., Heath S., Mamasoula C., Cosgrove C., Blue G., Granados-Riveron J., Setchfield K. (2013). Genome-wide association study of multiple congenital heart disease phenotypes identifies a susceptibility locus for atrial septal defect at chromosome 4p16. Nat. Genet..

[48] Hinrichs A.S., Karolchik D., Baertsch R., Barber G.P., Bejerano G., Clawson H., Diekhans M., Furey T.S., Harte R.A., Hsu F. (2006). The UCSC Genome Browser Database: update 2006. Nucleic Acids Res..

[49] Jiang X., Yan J., West A.A., Perry C.A., Malysheva O.V., Devapatla S., Pressman E., Vermeylen F., Caudill M.A. (2012). Maternal choline intake alters the epigenetic state of fetal cortisol-regulating genes in humans. FASEB J..

[50] Chan J., Deng L., Mikael L.G., Yan J., Pickell L., Wu Q., Caudill M.A., Rozen R. (2010). Low dietary choline and low dietary riboflavin during pregnancy influence reproductive outcomes and heart development in mice123. Am. J. Clin. Nutr..

[51] Chowdhury S., Hobbs C.A., MacLeod S.L., Cleves M.A., Melnyk S., James S.J., Hu P., Erickson S.W. (2012). Associations between maternal genotypes and metabolites implicated in congenital heart defects. Mol. Genet. Metabol..

[52] Tatara M., Ikeda T., Namekawa S.H., Maezawa S. (2023). ATAC-Seq Analysis of Accessible Chromatin: From Experimental Steps to Data Analysis. Methods Mol. Biol..

[53] Wenz B.M., Dudek M.F., Ramdas S., Creasy K.T., Xin D., Olthoff K.M., Shaked A., Rader D.J., Brown C.D., Voight B.F. (2025). Expanded Chromatin Accessibility Mapping Explains Genetic Variation Associated with Complex Traits in Liver. medRxiv.

