# Supplementary material for: Identifying genes associated with obstructive congenital heart defects using a family-based genetic random field method
Source: HGG Adv. 2026 Jul 6;7(4):100648. doi: 10.1016/j.xhgg.2026.100648 (PMC13445198; doi:10.1016/j.xhgg.2026.100648)

HGGA, Volume *7*

Supplemental information

Identifying genes associated with obstructive congenital heart defects using a family-based genetic random field method

Manyan Huang, Nianjun Liu, Stephanie M. Ware, John S. Witte, Bruce D. Gelb, Wendy N. Nembhard, Martha Werler, Meredith M. Howley, Lorenzo Botto, A.J. Agopian, Omobola O. Oluwafemi, Mary M. Jenkins, Lynn M. Almli, Andy Olshan, Paul A. Romitti, Gary Shaw, Ming Li, and the National Birth Defects Prevention Study

Supplemental Materials

Table S1. Genomic regions and nearby genes associated with congenital heart defects with p-value < 10^{-5}, the National Birth Defects Prevention Study, 2003-2011

	Chr	Regions	Gene	p-value									# of SNPs in region	
				Common variants analysis			Rare variants analysis			All variants analysis				
				Phase I	Phase II	Fisher	Phase I	Phase II	Fisher	Phase I	Phase II	Fisher	Common	Rare
FGRF-O	1	120161999-120190390	*ZNF697*	7.56×10^{-1}	1.32×10^{-1}	3.30×10^{-1}	4.66×10^{-1}	3.16×10^{-8}	2.80×10^{-7}	3.60×10^{-1}	2.90×10^{-7}	1.78×10^{-6}	36	34
FGRF-O	6	158589378-158620376	*GTF2H5*	2.87×10^{-6}	5.26×10^{-1}	2.17×10^{-5}	6.65×10^{-1}	3.82×10^{-1}	6.02×10^{-1}	2.99×10^{-1}	5.79×10^{-1}	4.77×10^{-1}	38	34
FGRF-O	6	158530535-158589312	*SERAC1*	7.92×10^{-7}	3.76×10^{-1}	4.78×10^{-6}	4.80×10^{-1}	8.29×10^{-1}	7.64×10^{-1}	2.00×10^{-1}	>0.999	5.22×10^{-1}	35	50
FGRF-O	7	100318422-100321323	*EPO*	1.82×10^{-1}	2.37×10^{-1}	1.79×10^{-1}	6.70×10^{-6}	3.34×10^{-1}	3.14×10^{-5}	3.18×10^{-4}	4.07×10^{-1}	1.29×10^{-3}	9	8
FGRF-O	15	48095580-48138433	*LINC01491*	5.22×10^{-1}	7.97×10^{-1}	7.81×10^{-1}	2.42×10^{-5}	>0.999	2.81×10^{-4}	4.92×10^{-6}	9.17×10^{-1}	6.00×10^{-5}	59	50
FGRF-F	11	125440185-125462463	*STT3A-AS1*	2.99×10^{-4}	1.48×10^{-3}	6.93×10^{-6}	4.00×10^{-1}	7.90×10^{-1}	6.80×10^{-1}	6.74×10^{-2}	5.33×10^{-1}	1.56×10^{-1}	23	12
FGRF-F	19	44978644-45004575	*ZNF180*	7.62×10^{-1}	8.50×10^{-6}	8.39×10^{-5}	5.69×10^{-1}	5.98×10^{-2}	1.49×10^{-1}	5.90×10^{-1}	8.44×10^{-2}	1.99×10^{-1}	41	43
FGRF-F	19	10713120-10755235	*SLC44A2*	4.56×10^{-1}	4.93×10^{-1}	5.60×10^{-1}	3.58×10^{-8}	2.27×10^{-2}	1.79×10^{-8}	6.31×10^{-14}	2.21×10^{-4}	5.55×10^{-16}	13	24
FGRF-F	19	51774519-51784488	*LINC01872*	1.83×10^{-1}	3.30×10^{-1}	2.30×10^{-1}	2.29×10^{-4}	4.39×10^{-1}	1.03×10^{-3}	4.60×10^{-6}	4.73×10^{-1}	3.05×10^{-5}	12	23

Abbreviations: Chr, chromosome; SNV, single nucleotide variant; FGRF-O, family-based genetic random field, overall test; FGRF-F, family-based genetic random field, Fisher's combined probability test.

Table S2. Coordinates of significant genomic regions in GRCh37/hg19 and their corresponding coordinates and gene annotations in GRCh38/hg38.

Gene	Gene coordinates (GRCh37/hg19)	LiftOver coordinates (GRCh38/hg38)	Gene coordinates (GRCh38/hg38)
ZNF697	chr1:120161999-120190390	chr1:119619376-119647767	chr1:119619377-119648266
SERAC1	chr6:158530535-158589312	chr6:158109503-158168280	chr6:158109519-158168280
SLC44A2	chr19:10713120-10755235	chr19:10602444-10644559	chr19:10602457-10644557

Table S3. Variants within gene *SLC44A2* and their testing results via single-variant-based transmission disequilibrium test (TDT).

Variant ID	Chr	BP	Allele1	Allele2	Phase I			Phase II		
					MAF	OR	p-value	MAF	OR	p-value
rs11665795	19	10706377	T	C	0.2349	1.013	0.909	0.2703	0.8547	0.2485
kgp1569424	19	10707400	T	G	0.07306	1.017	0.9263	0.06499	1.29	0.2855
kgp3562993	19	10707459	T	C	0.01167	2.111	0.05878	0.01721	0.4615	0.1083
kgp3801749	19	10708593	A	T	0.4721	0.9851	0.8809	0.4334	1.122	0.3701
kgp7078809	19	10708973	A	G	0.02789	0.7273	0.3304	0.01778	0.8333	0.6698
kgp7481012	19	10709991	A	C	0.03941	1.156	0.5472	0.02739	1.286	0.4795
kgp2296245	19	10710412	C	T	0.3271	0.9579	0.6783	0.357	0.9524	0.7021
kgp29556343	19	10711393	G	A	0.001484	0	0.0455	0.00353	NA	0.0455
kgp11993829	19	10714058	G	T	0.03883	0.6452	0.1235	0.03526	0.6087	0.139
rs12982370	19	10715154	A	C	0.319	0.9945	0.9581	0.3456	0.9752	0.8461
kgp543594	19	10719055	A	G	0.006197	2	0.2482	0.01283	0.6667	0.6547
kgp7909803	19	10724441	G	T	0.08704	0.9577	0.7991	0.07628	1.056	0.8162
kgp21358869	19	10727925	C	T	0.00173	0	0.08326	0.002646	NA	0.3173
kgp2878641	19	10731745	T	C	0.1973	0.9137	0.4619	0.1874	1.284	0.1245
kgp6199213	19	10732814	T	G	0.02533	0.7917	0.4458	0.03007	2.5	0.02334
kgp7475597	19	10733082	T	C	0.02984	1.941	0.02365	0.02881	1.188	0.6121
rs2360743	19	10736514	T	C	0.02512	0.0303	4.07E-08	0.01437	0	0.000312
exm1426459	19	10738415	C	T	0.000247	NA*	NA	0.000441	NA	NA
kgp29621429	19	10739531	G	A	0.007185	0.8	0.7389	0.01066	3.333	0.0522
kgp6020336	19	10739594	G	T	0.1304	1.154	0.3173	0.151	1.091	0.641
kgp430512	19	10741907	A	G	0.205	0.8562	0.182	0.1981	1.179	0.2829
rs7255431	19	10745923	T	C	0.002982	0.5	0.5637	0.002212	NA	NA
kgp21421290	19	10745942	A	C	0.003215	1	1	0.003965	NA	NA
kgp21357887	19	10745972	A	C	0.009886	0.3	0.0522	0.007489	1.5	0.6547
kgp21433636	19	10746205	A	G	0.009886	0.3	0.0522	0.007556	1.5	0.6547
kgp11126061	19	10746840	T	C	0.0302	0.875	0.6547	0.03348	0.7647	0.4652
kgp21399841	19	10748208	T	C	0.002475	0	0.3173	0.002657	3	0.3173
exm1426542	19	10748382	C	G	0.005958	0.3333	0.1573	0.007583	0.2	0.1025
rs3859514	19	10748820	T	C	0.3179	0.9031	0.3252	0.3282	1.124	0.3662
rs1560707	19	10750738	T	G	0.3181	0.8929	0.2756	0.3278	1.124	0.3662
rs10405617	19	10752968	A	G	0.2671	0.9883	0.9136	0.2784	1.15	0.3063
kgp21350773	19	10753449	C	T	0.002724	0.75	0.7055	0.002646	NA	0.3173
kgp21327895	19	10754303	C	T	0.002474	0	0.02535	0.000441	NA	NA
GA033957	19	10754814	A	G	0.04298	1.276	0.3248	0.04566	1.211	0.5371
GA033958	19	10755103	T	C	0.000497	NA	0.3173	0.001322	0.5	0.5637
kgp21484609	19	10759010	G	T	0.2699	0.9724	0.7913	0.2758	1.137	0.343
kgp6506546	19	10760704	C	T	0.009154	1.8	0.285	0.01058	0.5556	0.285

* Analysis conducted via plink1.9. OR and/or p-value cannot be calculated for some variants due to 0-count of transmitted or un-transmitted alleles.

Figure S1. UCSC Genome Browser tracks of the three significant gene-associated regions, showing RefSeq genes, ENCODE H3K27ac marks, and GM12878 Hi-C chromatin interactions for (A) *ZNF697*, (B) *SERAC1*, and (C) *SLC44A2*.

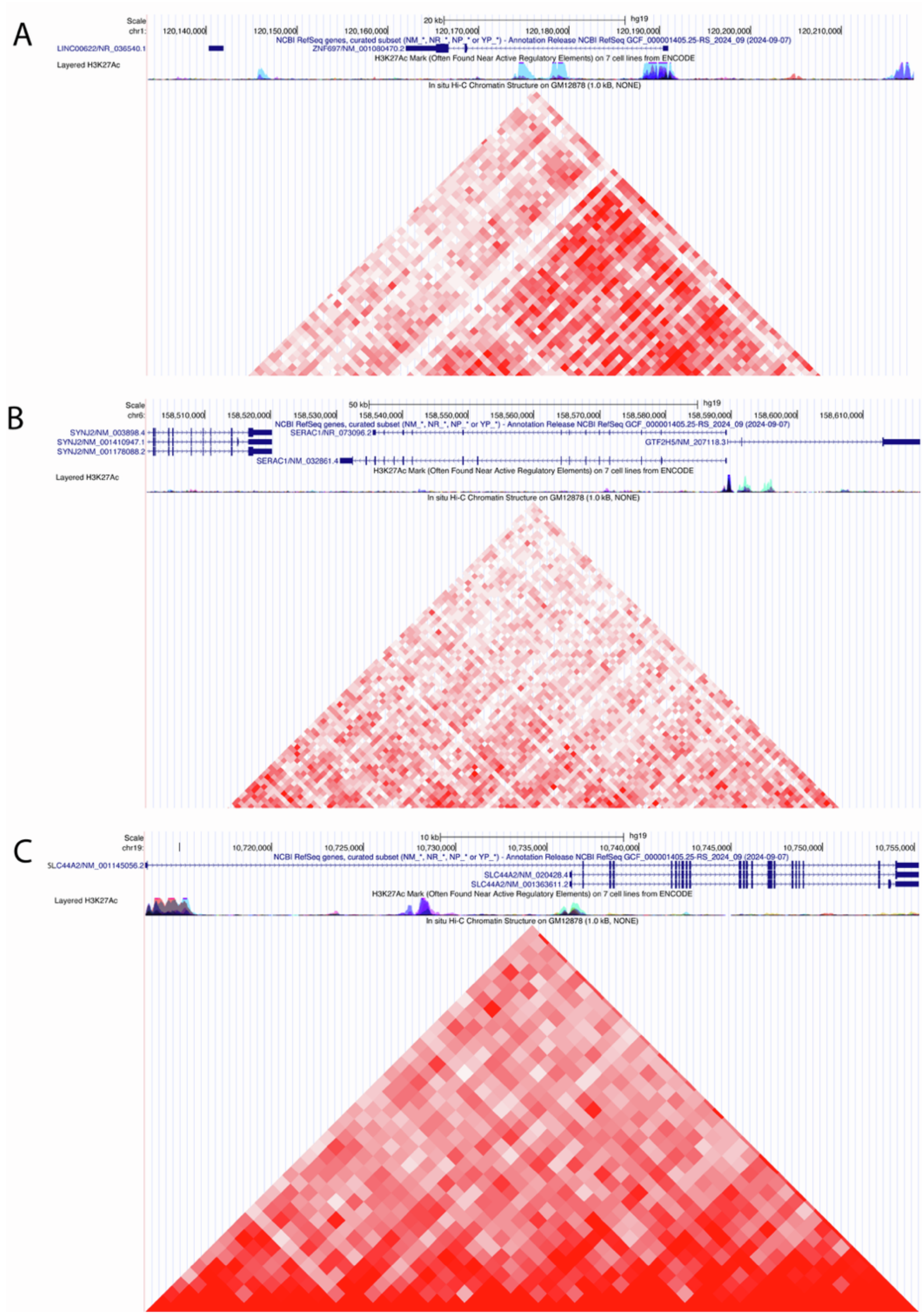

Supplement: Supplementary file 1 [file mmc1.pdf]
